# A review of ultrasound monitoring applications in agriculture

**DOI:** 10.3389/fpls.2025.1620868

**Published:** 2025-07-07

**Authors:** Muhammad Awais Sattar, Dina Shona Laila

**Affiliations:** Automatic Control, Department of Computer Science, Electrical and Space Engineering, Luleå University of Technology, Luleå, Sweden

**Keywords:** precision agriculture, ultrasound, nondestructive testing, sustainability, crop yield

## Abstract

Pursuing agricultural intensification to raise productivity has brought challenges such as involvement of high capitals, often in the form of loans, environmental damage, and ecosystem disruption. These challenges increase risks in agricultural practice that require good management and control. This increases the need for real-time, non-destructive monitoring technologies that can improve crop productivity, enhance land use, and facilitate environmentally friendly agriculture. Due to its unique capacity to non-destructively examine plants’ internal biological and structural properties, ultrasound has emerged as a promising non-invasive technique providing insights often unattainable with traditional optical, spectral, or chemical sensors. This review aims to provide an up-to-date state of the art in ultrasound-based monitoring applications within major agricultural areas: soil characterization, seed quality control, plant health, stress monitoring, pests and diseases detection, and fruit ripening assessment. This review explores how contact and non-contact ultrasound measurements are scalable and versatile, bridging the gaps between laboratory and field-deployed systems. Integrating ultrasound monitoring with artificial intelligence and Internet of Things (IOT) frameworks further enhances modality accuracy and can detect stress, diseases, and other physiological changes in crops sooner. Overcoming challenges such as environmental acoustic noise will require further work. Still, recent advances such as improved signal filtering algorithms, new transducer designs, better field sensitivity, and broader collaboration to standardize ultrasound measurement protocols indicate a growing trend toward increased on-field use of ultrasound. Finally, the review also discusses the current limitations and future research directions of how ultrasound-based monitoring can catalyse a new paradigm of sustainable data-driven agriculture that meets food security needs.

## 1 Introduction

Agriculture is the backbone of the global economy because it provides food, raw materials for industries, supports trade, generates employment, ensures food security, and drives economic development. In developing nations, agriculture is the largest source of livelihood (Ba, 2016). According to the estimates, agriculture employs around 1.3 billion people annually (Pandi and Shridar, 2017; Dedieu and Schiavi, 2019). Many research studies show that agriculture is vital in alleviating poverty and providing jobs (Singh et al., 2024; Chandrarekha et al., 2024). In recent years, climate change and an ever-growing demand for food production, among other factors, are increasing pressure on agriculture and threatening global food security and the sustainability of agricultural systems (Yu et al., 2025). The increasing temperatures, changes in precipitation patterns, droughts, floods, invasion of plant pests and diseases only add to the challenges for agricultural resilience. Simultaneously, the ever-increasing global population contributes to higher food demand, requiring the world to adopt sustainable agriculture practices (Hossain et al., 2024; Goswami et al., 2024; Rashmi et al., 2024).

To ensure global food security and economic growth, there is a constant need to improve the agricultural sector. Recent applications of advanced and digital sensing and control technology create what so called precision agriculture, aiming to enhance productivity and sustainability within the agricultural industry (Ambaru et al., 2025). Precision agriculture uses numerous cutting-edge technologies such as UAVs, machine learning techniques, and remote sensing to analyze the conditions of soil, livestock, and crops. The information obtained from these data-driven techniques can then be used to target interventions and decision-making at the farm level (Logeshwaran et al., 2024; Wang et al., 2024a; Xing and Wang, 2024; Agrawal and Arafat, 2024). Traditional methods for assessing plant health, such as chemical testing and manual inspection, have serious limitations, especially for farmers who need timely and accurate information to manage their crops. These approaches are labor-intensive, slow, destructive, and often produce inaccurate information, leading to delayed actions and potential yield losses (Ding et al., 2024; Fuentes-Peñailillo et al., 2024). Non-destructive testing (NDT) approaches present a promising alternative by providing real-time insights into plant health without causing harm.

In recent years, as NDT technologies continue to evolve rapidly, integrating them into existing agricultural practices could empower farmers to make data-driven decisions and increase their crop yields. These methods enable the early detection of disease and stress before a visible symptom appears. Additionally, they are low-cost solutions, making them more accessible to farmers for efficient crop management (El-Mesery et al., 2019; Mahanti et al., 2022; Egbokhaebho et al., 2023; Agarwal et al., 2024). Various NDT technologies have become highly useful in evaluating plant health, disease diagnosis, and monitoring while maintaining plant integrity. These technologies encompass diverse methods, from optical sensing, thermal cameras, and hyperspectral and multispectral analysis to acoustic and ultrasound approaches and fluorescence-based detection. Optical and spectral sensors help researchers monitor chlorophyll fluorescence, leaf reflectance, and changes in leaf pigmentation to detect early signs of physiological stress. Thermal imaging captures heat variations indicative of plant stress, and ultrasonic and acoustics techniques analyze internal structural conditions to aid in diagnosing structural weaknesses or early disease symptoms (Sinha et al., 2023; Joshi et al., 2024; Patel et al., 2022; Xu et al., 2024; Piriyadharshini and Ezhilarasi, 2023; Duveiller et al., 2024; Falcioni et al., 2024; Zhang et al., 2023; Wu, 2024). Among these NDT technologies, ultrasound has drawn increasing interest from researchers due to its ability to access internal plant structures, offer fast, non-destructive diagnostics, and detect early disease symptoms.

Ultrasound, a branch of acoustic wave technology, has emerged as a valuable imaging and sensing modality in medical and industrial fields primarily due to its non-destructive nature and ability to analyze biological tissues and materials. It enables real-time imaging without ionizing radiations in medical applications, a significant advantage over CT and X-ray imaging (Abdulsalam et al., 2023; Vrdoljak and Dravinac, 2025). Ultrasound is also being utilized for guided biopsies and other minimally invasive procedures. In recent advances, ultrasound molecular imaging is used for early cancer detection (Hashemi et al., 2024). Ultrasound is also widely used in structural health monitoring, food processing, crystallizations, multiphase flows, and mineral processing (Zhang et al., 2024; Liang et al., 2023; Baser et al., 2023; Wei et al., 2024; Nasir et al., 2024). Ultrasound has also proven to be a highly effective NDT tool in agriculture, offering unique advantages for plant health assessment and food quality assessment. Optical and thermal sensing methods only assess surface-level characteristics. Still, by utilizing high-frequency sound waves, ultrasound can penetrate the plant tissues and provide insights into their internal structure. It is particularly useful for detecting internal decay and changes in plant cell compositions. Advances in machine learning and signal processing have further enhanced the accuracy and usability of the modality in agriculture (Yan et al., 2024).

This review aims to provide a structured analysis of ultrasound monitoring in agriculture in the past decade. It discusses its core principles, such as acoustic properties, wave propagation, and diagnostic parameters like reflection, attenuation, and impedance changes. It further investigates ultrasound instrumentation and sensing techniques and explores the difference between contact and non-contact methods. It also explores imaging approaches like pulse-echo, through-transmission, and tomographic imaging. It further explores the role of ultrasound monitoring in major agriculture applications, such as soil analysis, seed quality assessment, plant health monitoring, pest and disease detection, and fruit ripeness evaluation. This review will discuss recent advancements in ultrasound monitoring, such as AI integration, IoT-enabled ultrasound sensors, and multi-modal diagnostic ultrasound systems deployed in agriculture.

## 2 Principles of ultrasound monitoring

**Figure 1:**
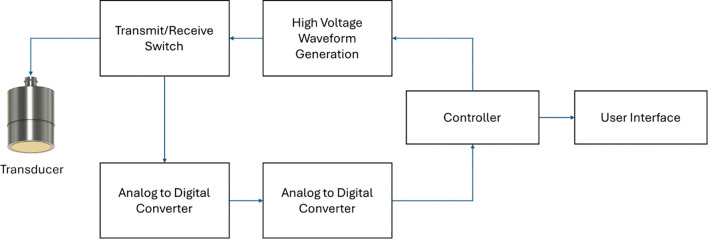
Schematic of a typical ultrasound system adapted from (Kidav et al., 2022; Chen and Pertijs, 2021).

Ultrasound systems send high-frequency acoustic waves to the material and analyze their interaction with it. Ultrasounds are mechanical pressure waves above 20 kHz, often in the MHz range for ultrasound imaging (Oates, 2023). The speed *c* of ultrasound waves in a medium can be determined by its inertial and elastic properties. The wavelength *λ* can be estimated by Equation 1:


(1) $$\text{λ}=\frac{c}{f},$$

where *f* is the frequency. As the equation shows, higher frequencies result in shorter wavelengths, leading to higher potential resolution. An ultrasound system consists of several components that generate data and images. It includes a pulser or transmitter that activates the transducer to emit ultrasound waves. A transmit/receive switch manages the signal flow between transmission and reception. The analog front end processes the received signal before converting it into digital form using an analog-to-digital converter. Finally, a processing unit further enhances the data, visualizing the ultrasound data/image on the user display (Kidav et al., 2022; Chen and Pertijs, 2021). A typical schematic of an ultrasound system is shown in **Figure 1**.

### 2.1 Wave propagation, acoustic impedance, and attenuation

Ultrasound waves travel through mediums, reflecting and transmitting at the boundaries where the material properties of the medium change. This behavior is determined by the acoustic impedance, which is defined by Equation 2:


(2) Z=ρc.

The density *ρ* and speed of sound *c* determine the proportion of an ultrasound wave that is reflected vs. transmitted at a boundary (Pusppanathan, 2017). A significant impedance mismatch causes strong reflections. For example, when an ultrasound wave traveling in a tissue encounters air, the impedance difference causes nearly total reflection, resulting in minimal transmitted energy (Grager et al., 2018). Medical ultrasound imaging maps the reflection from the tissue interfaces of differing impedance. For normal incidence, the reflection coefficient *R*, is defined as the ratio of reflected incident pressure amplitude defined as Equation 3:


(3) $$R=\frac{Z_{2}-Z_{1}}{Z_{2}+Z_{1}}.$$

As (3) shows, a greater contrast in acoustic impedance *Z* results in a stronger echo (Yared, 2011). This is essential in both medical and industrial applications. In medical applications, echoes form an image and in industrial applications, echoes help detect flaws (Martynenko and Ermachenko, 2021; Zhong et al., 2022). The intensity of ultrasound waves is attenuated as they travel through a medium. This attenuation or loss occurs due to absorption, scattering, and reflection, and is defined exponentially as Equation 4 (Zheng et al., 2024):


(4) $$I\left(x\right)=I_{0}e^{-2\alpha x}.$$

It means that the intensity decreases with distance. In soft tissues, attenuation typically increases linearly with the frequency (often 0.5 dBcm^−1^ MHz^−1^) as a rule of thumb. Thus, higher-frequency waves penetrate a shorter distance while offering higher resolution. By contrast, a lossless medium like water has negligible attenuation, and many solids (metals) have low intrinsic absorption. However, scattering from the microstructure can attenuate the wave. Gases cause very high attenuation of ultrasound, especially at high frequencies (Gudra, 2008; Su et al., 2017; Wells and Liang, 2011).

### 2.2 Ultrasound transducers and signal generation

**Figure 2:**
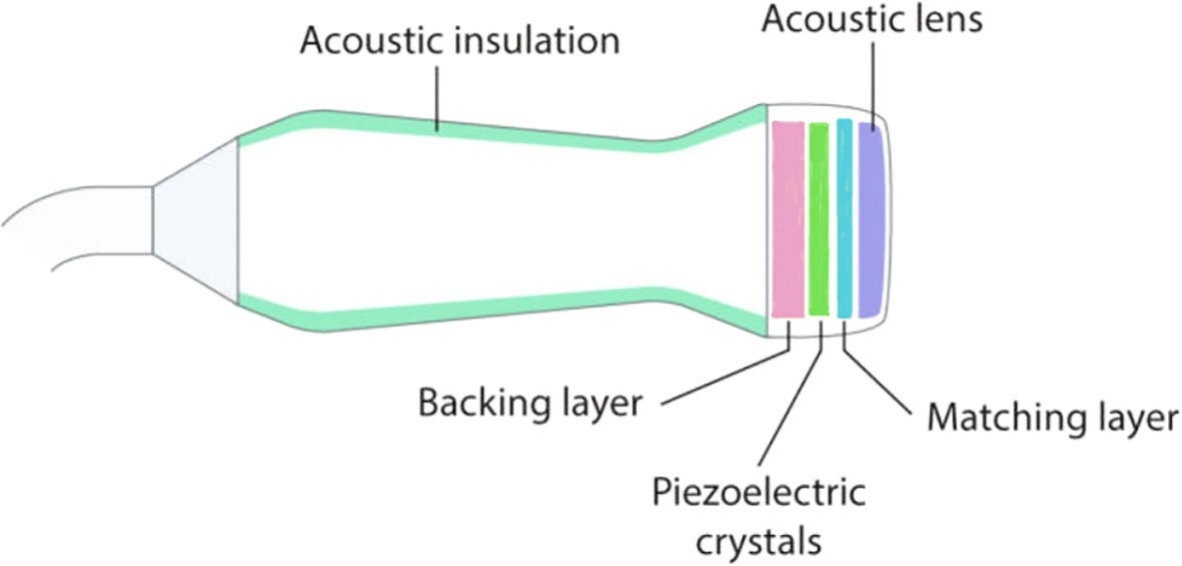
Schematic of a medical ultrasound transducer (Ricci et al., 2024).

The transducer is the most critical component in an ultrasound system. It converts electrical energy into acoustic waves and vice versa. Most ultrasound transducers are piezoelectric. A driving voltage pulse causes the piezoelectric crystal to vibrate mechanically, launching an acoustic wave (He et al., 2023). Upon receiving an echo, the pressure wave deforms the crystal, generating an electrical signal. Transducers are engineered with several key components: a piezoelectric element, a backing material, and one or more matching layers on the emitting face. The backing dampens the vibration duration (producing a broadband pulse) and reduces ringing. The acoustic matching layer is a critical design feature to efficiently transfer energy into the load medium, typically a quarter-wavelength thick layer with acoustic impedance intermediate between the high-impedance crystal and the lower-impedance medium (Toffessi Siewe et al., 2023; Barakat, 2023; Lu et al., 2023). A schematic illustration of an ultrasound transducer is shown in **Figure 2**.

Transducer frequency is selected based on the application. Medical probes for abdominal imaging operate around 1–5 MHz, whereas intravascular ultrasound or ophthalmic probes may use 20–60 MHz for fine resolution (Li et al., 2010; Luca et al., 2021). In industrial testing, lower frequencies (0.1–5 MHz) are standard for thicker or more attenuating materials, whereas higher frequencies (10–20 MHz) are used for fine-grained materials or thin parts (Krautkrämer and Krautkrämer, 1990). Because ultrasound does not travel efficiently through air, a coupling medium is typically required for contact transducers. In medical imaging, a gel is applied between the probe and the skin to displace air. In industrial inspections, liquid couplants (e.g., glycerin, oils) or water-immersion setups are used (Cheng et al., 2022; Koulountzios et al., 2019). On the other hand, non-contact methods (discussed below) avoid liquid couplant by generating or detecting ultrasound through air or electromagnetic waves.

### 2.3 Contact vs. non-contact ultrasound techniques

Contact ultrasound is the default approach in medical diagnostics and many NDT applications since it allows efficient acoustic coupling. A couplant (gel or liquid) is applied to minimize the air gap and reduce reflection losses at the interface. This yields a strong signal and high signal-to-noise ratio. Non-contact ultrasound is required where the direct coupling is infeasible or undesirable (e.g., hot surfaces, moving parts, large-scale scanning). The most common approach is air-coupled ultrasound. However, due to the severe impedance mismatch between typical transducer materials and air, only a tiny fraction of energy couples into air. Specialized air-coupled transducers are designed with lower frequencies to mitigate attenuation, high driving voltages and sensitive detection (Liu and Abdulla, 2023; Bente et al., 2023).

Other, non-piezoelectric, transducers such as capacitive micromachined ultrasonic transducers and optical/laser-based approaches have also advanced, permitting non-contact measurement with broader bandwidth or higher sensitivity (Yuan et al., 2023; Song et al., 2015). Electromagnetic acoustic transducers provide a non-contact option on conductive materials. They induce ultrasonic waves by electromagnetic forces in the test object (Jiang et al., 2023). However, EMATs efficiency can be lower than piezoelectric transducers, requiring powerful pulsed excitation. Another non-contact technique is laser ultrasound, where a pulsed laser generates ultrasonic waves via thermal expansion or ablation, and a separate interferometric laser detects surface displacements (Narumanchi et al., 2023). This is couplant-free and can operate at a standoff distance, but requires expensive, sensitive optical equipment. A comparison between contact and non-contact ultrasound is shown in **Table 1**.

**Table 1:** Comparison between contact and non-contact ultrasound techniques.

Feature	Contact ultrasound	Non-contact ultrasound
Resolution	High resolution due to direct contact	Lower resolution due to impedance mismatch
Penetration	Good penetration depth	Limited penetration depth
Coupling Medium	Requires a coupling medium (e.g., gel)	No coupling medium required
Applications	Widely used in medical imaging	Used in industrial and specialized medical cases
Advantages	High image quality, widely applicable	Non-invasive, suitable for sensitive areas
Limitations	Requires skin contact, may not be suitable for open wounds	Lower resolution, limited penetration

### 2.4 Ultrasound imaging and measurement technique

**Figure 3:**
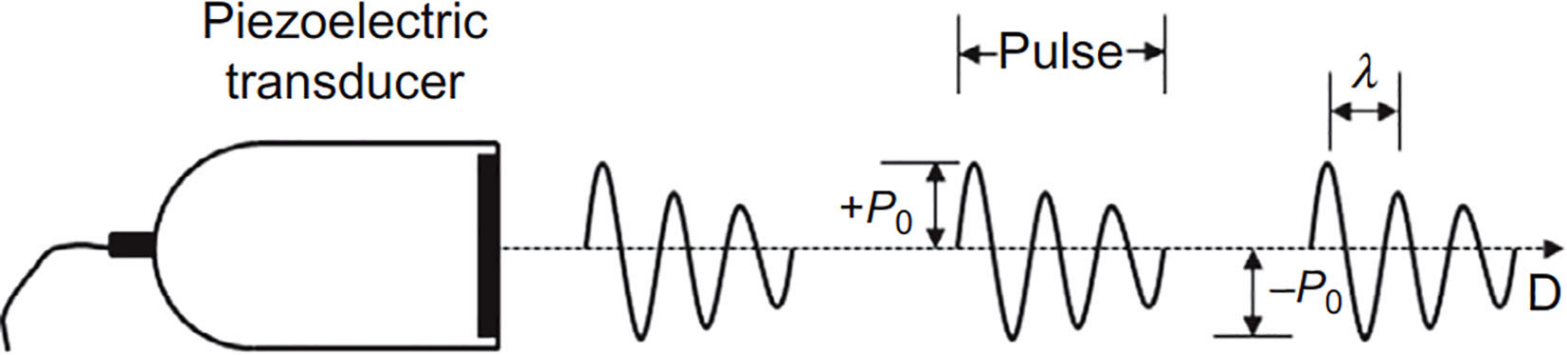
Illustration of the pulse-echo mode in ultrasound (Sennoga, 2020).

Depending on the application, various ultrasound modes, such as pulse-echo, through-transmission, and tomography, are utilized to gain in-depth information on the material under observation (Zhang and Cegla, 2022; Singh et al., 2022). Each technique differs in its operational concept, transducer arrangement, and data collection mechanism, providing distinct application advantages. In pulse-echo mode, a transducer sends short ultrasonic pulses (**Figure 3**) into the material and receives the echoes that return. The system measures time-of-flight and echo amplitude to measure internal features (Yanez et al., 2022). This is fundamental to both medical B-mode imaging and nondestructive flaw detection. Modern array-based systems can perform beamforming by introducing electronic delays, improving lateral resolution, and enabling scanning without physically moving the probe (Foiret et al., 2022).

**Figure 4:**
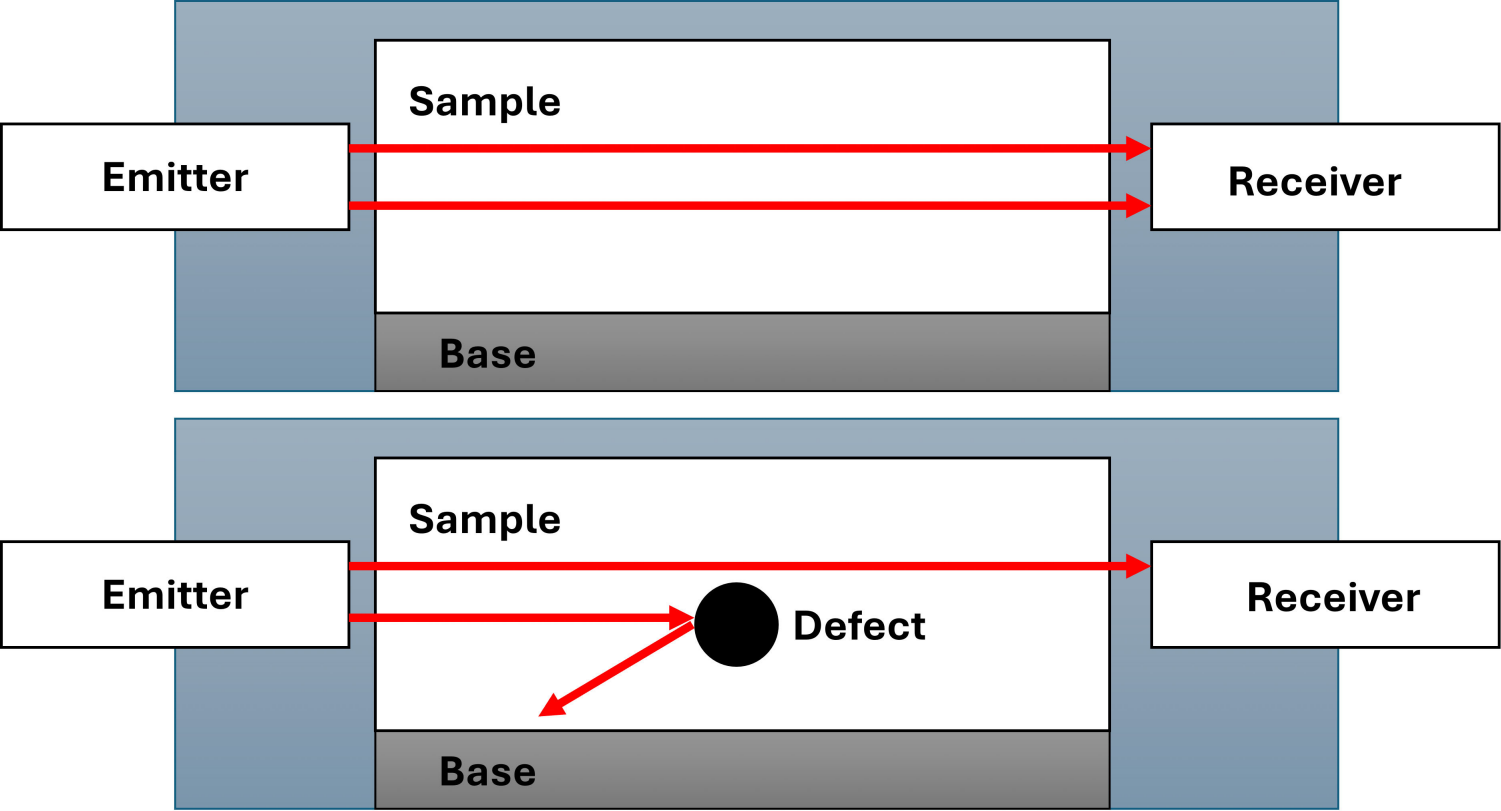
Illustration of the through transmission mode in ultrasound (Huang et al., 2023).

The through transmission mode places a transmitter on one side of the object and a receiver on the opposite. Instead of echoes, it measures how much ultrasound wave passes through, indicating attenuation or disruptions (like flaws) in the material, as shown in **Figure 4**. This method requires access to both sides but can yield direct measurements of transmitted intensity (Lluveras Núñez et al., 2017). It is often used to detect large internal voids or regions of high attenuation. Depth information is not directly obtained unless combined with scanning or tomography.

Tomographic methods reconstruct 2D or 3D maps of acoustic properties by combining multiple measurements from different angles. In transmission tomography, one measures the time-of-flight and attenuation along numerous paths around the target, then uses inverse algorithms to compute spatial distributions of sound speed and attenuation (Wiskin et al., 2019). Reflective tomography collects echo data from multiple vantage points, similar to seismic imaging, to build a reflectivity map (Zhang et al., 2018). Hybrid methods capture both transmitted and reflected signals. A classic medical example is breast ultrasound computed tomography, where an array encircles the breast in a water tank, sequentially emitting pulses and recording transmitted/reflected waves in all directions. Industrially, ultrasonic tomography can be used for pipeline or structural inspections by placing multiple transducers around a test object and reconstructing internal features from the measured signals (Lyu et al., 2024).

## 3 Ultrasound in agriculture

This section presents applications of ultrasound monitoring in agriculture. It is organized into the following subsections: Subsection 3.1 discusses applications in soil monitoring; Subsection 3.2 focuses on seed quality monitoring; Subsection 3.3 explores plant health monitoring; Subsection 3.4 examines pest and disease monitoring; and Subsection 3.5 covers fruit ripeness monitoring.

### 3.1 Soil monitoring

Ultrasound waves are utilized in soil monitoring applications to assess soil properties such as moisture content, texture, porosity, and structural integrity. To determine the metal concentration in soil samples, (da Silva Medeiros et al., 2020) utilized ultrasound-assisted extractions. Cavitations induced by the ultrasound accelerated the disintegration of soil particles, promoting the release of trace metals like Aluminum, Cadmium, Copper, Nickel, and Zinc. This method is efficient, straightforward, and environmentally friendly compared to the traditional acid digestion in monitoring. In another study, (Wang et al., 2024b) studied the propagation mechanism of ultrasound waves at the transducer-soil interface. By examining excitation frequency and amplitude, this study revealed the mechanism of energy transport inside the soil. The result provided a foundation for creating ultrasonic soil sensors and enhancing *in-situ* soil evaluation. (Woo et al., 2022) used a noncontact ultrasound system to measure the moisture in the soil. Ultrasound obtained the variation in soil strength and moisture accurately across sand, silt, and clay. It also employed a machine learning approach to predict moisture levels. In another work, (Orhan et al., 2022) developed a digital ultrasound-based soil texture analyzer that estimates silt, sand, and clay content in a soil-water mixture. The system eliminates the need for soil analysis in labs and offers low-cost and portable solutions for texture analysis in agricultural applications. This was further improved by incorporating pH, electrical conductivity data, and machine learning (Kilinc and Orhan, 2025). Bradley and Ghimire (2024) designed a noncontact ultrasound system to measure the soil porosity. This approach determined the porosity with high precision, and the technique was validated for dry agricultural soils. (Xu et al., 2023) used ultrasound wave velocity measurements to access the efficacy of microbially induced carbonate precipitation in stabilizing shale soils. The wave velocity showed a correlation with unconfined compressive strength and CaCO_3_ content. This technique facilitated rapid and non-destructive assessment of soil enhancement. (Choi et al., 2021) focused on using ultrasound-assisted soil washing to treat heavy metals such as Cu, Pb, and Zn. Desorption with ultrasound and mixing yielded much higher efficiency than the traditional techniques. The technique worked best on smaller particles and under milder chemical conditions. However, significant degradation of PFAS was not observed, likely due to cavitation interference from soil particles. The summary is presented in **Table 2**.

**Table 2:** Summary of ultrasound-based soil monitoring techniques.

Authors (Year)	Technique used	How it works	Effectiveness
da Silva Medeiros et al. (2020)	Ultrasound-assisted extraction	Cavitation-enhanced metal extraction from soil.	Fast, efficient, and lowcost.
Choi et al. (2021)	Soil washing w/ultrasound	Ultrasound + mixing improves metal removal.	High efficiency, less chemical use.
Woo et al. (2022)	Contactless leaky Rayleigh waves	Surface waves track moisture.	*R* ^2^ 0.98, non-invasive.
Orhan et al. (2022)	Ultrasound-based texture analyzer	Intensity analysis of soilwater mix.	Portable, rapid soil texture analysis.
Kewalramani et al. (2022)	Dual-frequency PFAS removal	Desorption of PFAS from soil.	Limited degradation due to cavitation loss.
Xu et al. (2023)	Ultrasound for MICP	Velocity linked to CaCO_3_ in soil.	Strong UCS correlation.
Wang et al. (2024a, b)	Ultrasonic signal propagation in soil	Studies wave behavior at transducer-soil interface.	Foundational for soil instruments.
Bradley and Ghimire (2024)	Non-contact reflection	Reflected ultrasound estimates porosity.	Accurate within ±0.04.
Kilinc and Orhan (2025)	Ultrasound + EC/pH sensing	Combined input for ML-based texture prediction.	Better performance infield.

### 3.2 Seed quality monitoring

In the past, numerous studies have been conducted to assess the seed quality using ultrasound. To identify slight cracks that compromise the germination in cottonseed, (Zhang et al., 2022) utilized an air-coupled ultrasound system. The technique captured ultrasound echo signals from the cottonseeds, transformed them into color-encoded images, and classified them through deep learning models. This technique detected undetectable subtle damages via optical or thermal approaches with an average identification accuracy of 90.7%, (Jin et al., 2016) utilized an air-coupled ultrasound system coupled with principal component analysis (PCA) and K-nearest neighbor (KNN) classification to identify the worm or manually induced damage in corn seed. To collect the most helpful information, the ultrasound signals from both sides of the corn seed were collected and then reduced and deionized via PCA to extract the essential features. These features were then classified by using multiple pattern recognition algorithms. Among all the algorithms, KNN achieves the highest accuracy of 100% for the intact seeds and 97% for the damaged ones. In another study, (Guo et al., 2019) used an acoustic signal processing method based on Gaussian modeling and an improved extreme learning machine to estimate the damage in wheat kernels. The signals from sprout damaged, insect damaged and undamaged kernels were processed using a short-term Fourier transform and the Gaussian parameters were extracted. The technique showed a detection accuracy of 92% for undamaged, 96% for insect damage and 95% for sprout damaged kernels. The summary is presented in **Table 3**.

**Table 3:** Summary of ultrasound-based seed quality assessment techniques.

Authors (Year)	Technique used	How it works	Effectiveness
Jin et al. (2016)	Air-coupled ultrasound	Captures ultrasonic echo signals from corn seeds; features extracted and classified using pattern recognition algorithms (PCA + KNN).	High accuracy in classifying intact and damaged corn seeds; up to 100% for intact, 97% for damaged.
Zhang et al. (2022)	Air-coupled ultrasound with sound-to-image encoding	Encodes ultrasonic reflections from cottonseeds into RGB images; uses MobileViT-based deep learning model.	Accurate slight crack detection with 90.7% average accuracy; fast and non-destructive.
Guo et al. (2019)	Impact acoustic signal with Gaussian modeling and ELM	Uses impact-generated signals on wheat kernels; extracts features via time-frequency analysis and classifies with COAS-ELM model.	Non-contact detection with 95–96% classification accuracy for damaged kernels.

### 3.3 Plant health monitoring

Numerous studies have been done in recent years on plant health monitoring. Yang et al. (2022) utilized a non-contact ultrasound method called bulk modulus elastography to determine a cactus plant’s drying behavior and health status. The study utilized novel air-coupled transducers to capture the changes in the elastic modulus of prickly pear cactus (*Opuntia*) pad (nopal) over 11 days. This method can capture deep tissue changes and is also efficient in the early detection of stress conditions in agricultural contexts. (Gómez Álvarez Arenas et al., 2016) also used non-contact ultrasound to monitor changes in the leaf’s mechanical properties and determine the hydration condition of plants. The technique measured the change in the frequency of the leaf’s ultrasonic thickness, which is closely related to the relative water content and water potential. In the field experiments on the common grapevine (*Vitis vinifera*) and Arabica coffee (*Coffea arabica*) leaves, the system showed high sensitivity and repeatability in determining the draught and stress conditions in the plants. Similarly, to detect northern leaf blight in maize, (Maginga et al., 2024) utilized a non-contact ultrasound-based anomaly detection system integrated with IoT sound sensors. The method used the ultrasound emissions from maize stems, taking advantage of the fact that disease-induced stress alters the plant’s physiological acoustic signature. By training Long Short-Term Memory (LSTM) models on ultrasound data from healthy plants, the system was able to detect small deviations signaling early disease. The approach effectively detected disease 4–5 days before the visual symptoms with 99.98% accuracy.

Khait et al. (2023) used ultrasonic acoustic monitoring combined with machine learning to monitor the physiological conditions of tomato and tobacco plants. The experiments were conducted in the acoustic chamber and a greenhouse under drought and mechanical stress. The study revealed that the stressed plants emit species-specific and condition-specific airborne ultrasound sounds in the range of 20–100 Khz, which could be detected from 3–5 meter distance. Using a convolutional neural network (CNN) and support vector machine (SVM), the study successfully distinguished drought-stressed from the control plant with 84% accuracy and identified the dehydration levels with 81% accuracy. Khait et al. (2023) monitored changes in the thickness resonances of plant leaves using non-contact, air-coupled ultrasonic spectroscopy to determine the physiological responses of plants to environmental stimuli. In response to sudden watering after a drought, diurnal cycles, and rapid changes in light intensity, the system detected shifts in the resonant frequency (150–900 kHz). Leaf turgor pressure, relative water content (RWC), and tissue elasticity correlated with these frequency shifts. Wang et al. (2011) detected xylem cavitation events in tomato plants using an ultrasonic acoustic emission (UAE) monitoring system to determine the degree of plant water stress and support precision irrigation. Piezoelectric transducers incorporated into a multi-parameter system that measured temperature, humidity, CO_2_ concentration, light intensity, and transpiration rate were used to capture UAE signals in the 100 kHz to 1 MHz range. According to the study, the cumulative UAE signals showed clear diurnal patterns and a strong correlation with plant transpiration activity. Cavitation caused by water stress was reflected in the UAE peak’s slight lag the transpiration rate peak. Vergeynst et al. (2015) used broadband point-contact ultrasonic emission (UAE) sensing to investigate drought-induced cavitation and signal propagation in plant stems. The study looked at how ultrasonic signals travel from the source, such as xylem cavitation events, to the sensor by combining experimental detection of UAE signals in dehydrating branches with finite element modeling. This framework enables the differentiation of near- and far-field signals in woody species like *Vitis vinifera* and *Fraxinus excelsior* due to the AE source dynamics associated with abrupt xylem tension release. This method provides a high-resolution tool for monitoring xylem embolism under drought stress.

Charrier et al. (2015) used infrared thermography and UAE to study the ice nucleation and propagation in woody plants to investigate the response to freezing. The study found that the signals were strongest close to the nucleation point and decreased significantly with distance. This indicates a strong correlation with ice formation’s temporal and spatial dynamics. This work also concludes that the AEs were caused by cavitation events triggered by the tension at the ice–liquid interface, with the UAE source effectively following the moving ice front. This method is helpful for cold stress research and monitoring plant freezing resistance. To detect the internal and near-surface defect in trees, (Qiu et al., 2019) proposed a novel technique by integrating stress wave sensing with acoustics laser methods. Due to the limited propagation of surface waves, conventional sonic tomography has trouble identifying defects close to the bark. The authors solved this problem by combining data on stress wave transmission with measurements of surface vibration made with laser vibrometry, which measures variations in vibration amplitude caused by defects in the subsurface. The method was tested on *Cinnamomum camphora* trunks with 5–50 mm-deep artificial air holes and gaps. Internal (core) defects were clearly visible with conventional sonic tomography, but shallow defects (less than 25 mm deep) were not. Down to 5 mm from the surface, the integrated method successfully detected both internal holes and shallow defects, demonstrating significantly higher sensitivity and resolution. Bonisoli et al. (2025) used non-contact ultrasound to monitor plant drought stress in indoor and outdoor conditions. Tomato plants and pinto beans were subjected to water stress, and it was noticed that the stressed plants emit more ultrasound signals than hydrated controls. This study also considered the external noise factors, including wind, rain and insect chirping. The summary is presented in **Table 4**.

**Table 4:** Summary of ultrasound-based plant health monitoring techniques.

Authors (Year)	Technique used	How it works	Effectiveness
Wang et al. (2011)	Piezoelectric AE sensors + multi-parameter system	AE signal correlation with transpiration and cavitation	Drought stress monitoring
Fariñas et al. (2014)	Air-coupled ultrasound	Resonant frequency changes under light, drought, and diurnal cycles	Real-time water stress tracking
Charrier et al. (2015)	Acoustic emission sensors + IR thermography	AE tracking of ice propagation in xylem	Freeze stress monitoring
Vergeynst et al. (2015)	AE + waveform clustering + *µ*CT	AE waveform classification to distinguish cavitation sources	Hydraulic analysis under drought
Gómez Álvarez Arenas et al. (2016)	NC-RUS with broadband ultrasound	Non-contact resonant ultrasound to monitor leaf water status (RWC, Ψ)	Precision irrigation control
Qiu et al. (2019)	AE tomography + surface laser vibrometry	Integration of stress wave and acoustic-laser tomography	Structural tree health assessment
Yang et al. (2022)	Air-coupled ultrasound with raster scanning	Ultrasound elastography to monitor dehydration-induced modulus changes in cactus pads	Non-contact detection of tissue water loss
Khait et al. (2023)	Air microphones + ML classifiers	Airborne ultrasonic emissions from stressed plants	Plant stress class using AI classification
Maginga et al. (2024)	CNN-LSTM on ultrasound and VOC data	AI classification of plant stress from ultrasonic emissions and VOC data	IoT-based monitoring disease
Bonisoli et al. (2025)	Ultrasonic microphone + signal filtering	UE detection in outdoor conditions using microphones	Non-invasive detection in settings stress natural

### 3.4 Pest and disease monitoring

Ultrasound is conventionally defined as sound waves with frequencies above 20 kHz. However, in practical agricultural monitoring applications, systems operating just below this threshold, particularly in the 15–20 kHz range, are often included under ultrasound-based monitoring. This is because they utilize similar high-frequency sensor technologies, signal processing methods, and non-invasive approaches designed for detecting internal biological activity. Many insect species, especially larvae of soil- and wood-dwelling pests, produce informative signals such as stridulations and feeding sounds within this upper-audible to near-ultrasonic band. As a result, broadband acoustic systems spanning both audible and ultrasonic frequencies are commonly deployed. These systems are functionally aligned with ultrasound monitoring and are therefore considered relevant to the scope of this review (Mankin et al., 2011; Pinhas et al., 2008). In the past decade, many researchers have focused on the early detection of *Rhynchophorus ferrugineus*, also known as red palm weevil (RPW), a highly destructive pest of palm species. To analyze the ultrasound signals from RPW, (Martin et al., 2015) used an ultrasound-based acoustic technique to investigate the intensity and frequency of sounds emitted by RPW in coconut palms. The measurements were collected in both laboratory and field settings and further analyzed for distinctive spectral features. The system presented in this research did not give any real-time information, but the work laid the groundwork for the identification of pest-specific ultrasound patterns.

Hetzroni et al. (2016) used piezoelectric ultrasound sensors to detect larval chewing activity in date and canary palms. This study compared human and machine-assisted monitoring and demonstrated that both are feasible for detecting RPW in natural field conditions. The study also reported a human-assisted accuracy of 85% and a machine-assisted accuracy of 95%. Mankin et al. (2016) utilized advanced ultrasound signal processing to detect the RPW larval activity in commercial palm orchards. The study analyzed temporal and spectral ultrasound patterns to differentiate the environmental noise from the pest activity. The method was tested in the field and has shown capabilities in detecting multispecies of pests. To implement a scalable ultrasound-based monitoring system, (Ashry et al., 2022) introduced a distributed acoustic sensing (DAS) system that uses optical fiber to detect the ultrasound signals produced by RRW larvae. The system used phase-sensitive optical time domain reflectometry to detect the larval chewing activity in the 200–800 Hz frequency range. The system could capture RPW larval ultrasound emissions as early as 12 days post-infestation. The study also used a custom signal processing algorithm based on signal-to-noise ratio (SNR) to distinguish between infested vs healthy palm trees. The algorithm could detect 97 infestations in the infested trees, whereas only 9 in healthy trees. This system was further enhanced by deploying convolutional neural networks (CNN) in both controlled and outdoor farm environments. CNN was used to process the signals and classify them as infested’ or healthy. In the outdoor field trials the system showed an RPW larvae classification accuracy of 97%. Wang et al. (2021) also employed a fiber optic distributed acoustic sensing system integrated with machine learning to enhance early ultrasound-based detection of RPW in noisy farm environments. The system was used to record signals under various noisy conditions, and artificial neural networks (ANN) and CNN were trained to classify healthy vs infested trees. The study reported a classification accuracy of greater than 99% in combined noisy scenarios.

In addition to detecting RPW monitoring, researchers have also explored other agricultural pests. In coffee plantations, (Escola et al., 2020) developed a real-time acoustic detection system that utilized wavelet packet transform, bark scale filtering, and SVM classifications to detect and distinguish *Quesada gigas* cicadas signals from background noise. The study reported an accuracy of 96.41%. This work was further expanded by (de Souza et al., 2022) they integrated Paraconsistent Feature Engineering (PFE) and Empirical Mode Decomposition (EMD) for feature extraction, and the study reported classification accuracies of above 98% and reported the suitability of the system for smart farm deployment. In soil and root crop systems, (Görres and Chesmore, 2019) used acoustic monitoring system to detect the stridulation patterns of *Melolontha* and *M. hippocastani* larvae in soil. The study deployed a fractal dimension-based algorithm to distinguish stridulation events from the background noise. The automated analysis effectively detected the stridulation rates, which strongly correlated with the larval abundance. Nanda et al. (2023) used both acoustic and temperature signals to detect *Coptotermes curvignathus* in pine boards, which is considered one of the most damaging termites in Indonesia. The dual-sensing system used in the study was used to monitor real-time feeding, excavation, and alarm behavior. The study successfully detected the termite activity from the background noise over a 24-hour monitoring period. Additionally, the study also reported a rising temperature averaging 0.101 *°*C between healthy and infested samples. A regression model also confirms strong correlations between termite population and both temperature and acoustics signal duration. **Table 5** summarizes the work reviewed in this section.

**Table 5:** Summary of ultrasound-based plant disease and pest monitoring techniques.

Authors (Year)	Technique used	How it works	Effectiveness
Wang et al. (2011)	Ultrasonic xylem cavitation monitoring	Diagnosed xylem cavitation via acoustic emissions to assess drought-induced water transport failure.	Critical for drought stress physiology; early warning system for cavitation.
Fariñas et al. (2014)	Ultrasonic sensing of leaf response	Used ultrasound to monitor leaf reactions to environmental stimuli like drought or cold.	Effective for studying stress-response pathways in real time.
Charrier et al. (2015)	Ultrasound during ice propagation in xylem	Detected ultrasonic signals from ice formation in xylem during freezing.	Advanced understanding of frost damage mechanisms.
Vergeynst et al. (2015)	Acoustic emissions in drought-stressed branches	Linked acoustic emission signals to branch hydration status, distinguishing between sources.	Improved interpretation of drought-induced acoustic emissions.
Martin et al. (2015)	Acoustic RPW activity recording	Studied acoustic signatures of red palm weevil in coconut trees.	Useful for characterizing infestation patterns.
Gómez Álvarez Arenas et al. (2016)	Ultrasonic leaf sensing	Ultrasound sensors monitored plant water needs by detecting changes in leaf acoustics.	Highly sensitive to water stress; useful for irrigation scheduling.
Hetzroni et al. (2016)	Piezoelectric Acoustic RPW detection	Used piezoelectric sensors to detect RPW larvae in palms; machine vs human evaluation.	Machine accuracy 95%, human 85%; viable for field use.
Mankin et al. (2016)	Spectral-temporal acoustic analysis	Analyzed time-frequency patterns to detect RPW and *Oryctes elegans* in palm orchards.	Effective for multispecies detection in noisy conditions.
Qiu et al. (2019)	Acoustic-laser tomography	Used hybrid acoustic and laser method for defect detection in tree trunks.	Non-invasive, effective for structural assessment in forestry.
Görres and Chesmore (2019)	Fractal-based Acoustic larval detection	Detected stridulations of *Melolontha* larvae in soil via fractal dimension analysis.	Enabled species-specific, non-invasive larval monitoring.
Escola et al. (2020)	Acoustic + WPT + SVM	Used Bark scale filtering and wavelet transform to detect *Quesada gigas* in coffee crops.	Achieved 96.41% accuracy; low-cost, real-time solution.
Wang et al. (2021)	DAS + machine learning	Fiber optic DAS with CNN and ANN to detect RPW larvae in noise-rich environments.	Reported >99% accuracy in controlled noisy scenarios.
Yang et al. (2022)	Non-contact ultrasound in ambient air	Used ultrasound to monitor plant health responses without direct contact, detecting physiological changes remotely.	Promising for real-time monitoring in open-field conditions; non-invasive.
Ashry et al. (2022)	CNN-aided DAS via fiber optics	Used optical fiber DAS with CNN to detect early RPW infestation from chewing sounds.	Achieved ∼97% classification accuracy in field tests.
(de Souza et al., 2022)	EMD + PFE for cicada detection	Applied empirical mode decomposition and paraconsistent logic for robust signal classification.	Reached >98% accuracy; low computational load for field use.
Khait et al. (2023)	Airborne stress-induced sound recording	Captured airborne ultrasound-like sounds emitted by stressed plants, confirming they are informative.	Revealed plants emit detectable sounds under stress, usable for remote stress sensing.
Nanda et al. (2023)	Acoustic + thermal termite monitoring	Monitored termite activity in wood using acoustic (22 kHz) and temperature sensors.	Successfully correlated temperature and activity; avg. temp rise 0.101C.
Maginga et al. (2024)	Ultrasound IoT + CNN-LSTM	Combined wavelet transform and deep learning on ultrasound & VOC data for maize disease detection.	High accuracy in nonvisual early disease identification.
Bonisoli et al. (2025)	Contactless plant ultrasonic monitoring	Outdoor detection of plant ultrasound emissions using microphones without contact.	Suitable for field applications; validates airborne acoustic sensing.

### 3.5 Fruit ripeness monitoring

Several studies in the literature highlight the usage of ultrasound in monitoring fruit ripeness. This section will explore studies done in the last decade. Miraei Ashtiani et al. (2016) used ultrasonic spectroscopy to determine postharvest changes in the chemical and mechanical properties of the persimmons fruits. The study used Teflon-coated ultrasonic transducers to monitor ultrasonic velocity and attenuation as the fruit ripened over 21 days. These parameters were further statistically modeled with the coefficient of determination (*R* ^2^) value greater than 0.82 to predict the modulus of elasticity, rupture force, and soluble solid content (SSC) that indicates the ripeness and quality of the fruit. Vasighi-Shojae et al. (2018) used a portable ultrasonic system to determine apples’ firmness, rupture energy, and elastic modulus. This study measured the velocity and attenuation through the entire fruit and developed multiple linear regression models to predict the properties of the fruit. The model demonstrated a promising predictive capability with *R* ^2^ values up to 0.73, indicating moderate to high correlation. This technique was further enhanced by combining ultrasonic measurements with artificial neural networks (ANN) by (Vasighi-Shojae et al., 2020). Using the measured acoustic properties of apples, ANN models were developed to predict firmness, elastic modulus, and stiffness. The models developed in this work demonstrated a high predictive accuracy with *R* ^2^ = 0.99 for all three properties.

Soltani Firouz et al. (2021) used a custom ultrasound system together with a support vector machine (SVM) classifier to determine the mechanical effects of freeze in oranges. Ultrasonic velocity and attenuation were measured through the fruit, and the impact of freezing was analyzed. The study found that the freezing altered the fruit’s internal properties, affecting ultrasound propagation, which can be detected immediately after freezing and before the visual symptoms appear. Using SVM classifiers, the system achieved an accuracy of 100% in distinguishing healthy, mildly damaged, and severely freeze-damaged oranges. Budiastra and Jannah (2022) utilized ultrasonic methods to determine the firmness and sweetness of soursop fruit. Velocity and attenuation were measured using the ultrasound system, which showed a strong correlation, with physicochemical parameters firmness having *R* ^2^ = 0.884 and total soluble solids (TSS) having *R* ^2^ = 0.81. In the study, two regression models were also developed to classify firmness and sweetness levels, with a classification accuracy of 100% and 95%, respectively, when validated against manual measurement on 20 randomly selected samples. **Table 6** summarizes the work reviewed in this section.

**Table 6:** Summary of ultrasound-based fruit quality assessment techniques.

Authors (Year)	Technique used	How it works	Effectiveness
Miraei Ashtiani et al. (2016)	Ultrasonic spectroscopy	Measures ultrasonic velocity and attenuation through persimmons during ripening; uses regression models to predict mechanical and chemical attributes.	R^2^ *>* 0.82 for firmness, elasticity, and SSC; effective and accurate for non-destructive ripeness assessment.
Vasighi-Shojae et al. (2018)	Ultrasonic Velocity and attenuation	Uses 40 kHz ultrasound through apples; features fed into regression to predict internal quality traits.	Moderate to high prediction accuracy (R^2^ = 0.73); feasible for real-time monitoring.
Vasighi-Shojae et al. (2020)	Ultrasound with Artificial Neural Network	Measures ultrasonic parameters from apples and uses ANN models to predict firmness, modulus, and stiffness.	Very high accuracy (R^2^ = 0.999); ANN models outperform regression for mechanical property prediction.
Soltani Firouz et al. (2021)	Low-intensity ultrasound + SVM	Measures changes in ultrasonic propagation in oranges before and after freezing; uses SVM classifier.	Achieves 100% accuracy in classifying freeze damage severity; enables early detection before symptoms appear.
Budiastra and Jannah (2022)	Throughtransmission ultrasound	Applies 50 kHz ultrasound to soursop fruits; velocity data used in regression models for firmness and sweetness.	Classification accuracy of 100% for firmness and 95% for sweetness; non-invasive and robust.

### 3.6 Commercially available ultrasound systems for agricultural use

Several specialized ultrasound-based systems are commercially available for in-field crop and tree monitoring. For the structural health of trees, sonic tomograph devices are used to detect internal decay or cavities non-invasively. Notably, the PiCUS Sonic Tomograph (Argus Electronic GmbH, Germany) (IML Electronic GmbH, 2021) and ArborSonic 3D (Fakopp Enterprise, Hungary) employ multiple contact piezoelectric sensors around a trunk to measure sound wave transit times and construct cross-sectional images of wood integrity (Fakopp Enterprise Bt, 2023). Similarly, the Arbotom system (Rinntech, Germany) (Rinntech GmbH, 2022) uses impulse sonic tomography to map tree trunk and limb quality for risk assessment. These systems are field-portable and designed for outdoor use by arborists or growers to evaluate trunk and canopy structural health (e.g., detecting hollows, rot, or cracks) without harming the tree.

Ultrasound sensing has also been applied to pest and stress monitoring. For example, IoTree sensors (Agrint, Israel) are wireless in-tree seismic/ultrasonic devices that detect vibrations from wood-boring insect larvae (such as red palm weevil) inside live palm (Agrint Ltd, 2024). This contact sensor network is deployed in orchards and plantations, enabling early pest infestation detection under real field conditions. In the domain of plant physiological stress, emerging solutions like Plense Technologies’ system (Netherlands) use high-frequency acoustic sensors to “listen” to plants. Plense’s ultrasound sensor employs small ultrasonic microphones and speakers near the plant to detect xylem cavitation sounds and measure water content, providing real-time monitoring of drought stress in greenhouse or field (Plense Technologies, 2024). These examples illustrate the range of commercial ultrasonic technologies now available for in-field agricultural monitoring, from tree health diagnostics to pest detection and crop stress sensing. **Table 7** summarizes the commercially available devices.

**Table 7:** Commercially available ultrasound-based monitoring systems for field agricultural applications.

System (Manufacturer)	Application	Ultrasound sensing type and function	Reference
PiCUS Sonic Tomograph (IML Electronic, Germany)	Tree trunk decay detection	Uses contact piezoelectric sensors arranged around the trunk to measure sonic transit times and construct tomograms of internal wood structure.	(IML Electronic GmbH, 2021)
ArborSonic 3D (Fakopp Enterprise, Hungary)	Tree structural health	Employs multiple nail-mounted sensors for stress wave timing and 2D/3D acoustic tomographic imaging; detects hollows and decay.	(Fakopp Enterprise Bt, 2023)
Arbotom (Rinntech, Germany)	Tree integrity assessment	Uses impulse sonic tomography with a tapping hammer and contact sensors to map internal trunk condition.	(Rinntech GmbH, 2022)
IoTree (Agrint Ltd., Israel)	Insect pest detection in palms	In-tree seismic/ultrasonic sensor that detects larval vibrations (e.g., red palm weevil) and sends alerts wirelessly.	(Agrint Ltd, 2024)
Plense Ultrasound Sensor (Plense Technologies, Netherlands)	Plant physiological stress (e.g., drought)	Uses non-contact ultrasonic microphones and speakers to detect xylem cavitation signals and assess plant water status.	(Plense Technologies, 2024)

## 4 Discussion

Ultrasound-based sensing distinguishes itself from other precision agriculture tools by its depth of insight into agricultural applications. Unlike other commonly used modalities that can only capture surface characteristics, ultrasound waves can penetrate leaves, seeds, and soil layers, revealing internal states that would otherwise remain hidden. This ability to probe beneath the surface gives ultrasound a unique monitoring and diagnostic power. Studies showed that the ultrasonic measurements could reveal shifts in plant hydration and tissue elasticity that visual inspection alone could not detect. In practical terms, an ultrasonic sensor can act as a “stethoscope” for crops, listening for signs of stress within plant organs or soil profiles in real time. This non-destructive view of internal conditions avoids the cost and complexity of destructive sampling and positions ultrasound as an inexpensive, in-field enhancement to current precision agriculture technologies.

In soil applications, ultrasound provides real-time and non-destructive assessments of physical and chemical properties that are pivotal to the management of crops. For example, *in situ* soil moisture and texture measurements using ultrasonic waves provide rapid feedback on soil conditions, which would otherwise require lab analyses taking days or weeks. By measuring how quickly ultrasound travels through soil and how much it loses energy while doing so, these methods can estimate how much water there is in the soil or how tightly packed it is, information that guides irrigation and tillage choices. Ultrasound can even effectively assist in soil remediation efforts. It has been shown that ultrasound-assisted processes disintegrate soil aggregates and mobilize contaminants, enhancing the efficiency of heavy metal pollutant extraction from soil compared to conventional methods. Altogether, such soil-centric applications show ultrasound’s ability to facilitate soil health monitoring and remediation.

For seeds and fruit, the air-coupled ultrasonic imaging has been used on grains to search for microscopic internal cracks or insect damage that cuts the seed off from germination. When subjected to machine learning algorithms, such ultrasonic echoes allow the scanner to consistently separate healthy seeds from damaged ones, with over 90% (Jin et al., 2016; Guo et al., 2019; Zhang and Cegla, 2022) accuracy in crops such as cotton, corn, and wheat. Similar principles have been applied to fruit ripeness monitoring. The propagation speed and attenuation of ultrasonic waves through fruit tissue correlate with firmness and soluble solid content, essential indices for fruit ripeness, enabling growers to predict a proper harvest time non-invasively. Having ultrasound techniques like these helps maintain the valuable seed stock and the produce while still providing the vital quality information needed in the marketplace, instead of destructive testing.

Ultrasound can also keep a watch on the health of plants as they grow and alert scientists and farmers to the first sign of stress or sickness. Long before visible signs, studies tracked a cactus pad’s elastic modulus via ultrasound over several days of dehydration, revealing that the plant tissues react to water stress by changing their mechanical properties, and ultrasonic measurements can detect these changes. Similarly, the ultrasound of leaves may provide information about the state of the water. Changes in the frequency of ultrasonic vibrations passing through a leaf have been associated with loss of turgor and early drought stress in grapevine and coffee plants, respectively. Acoustic emissions in the ultrasonic range beyond drought can act as a warning indicating pathogenic attacks. In maize, IOT-connected ultrasonic sensors were placed on the stem of a plant and trained to use a deep learning model to recognize the acoustic signature of a fungal infection. This system detected a disease 4–5 days (Maginga et al., 2024) before it appeared on the leaves. Recent documentation of tomato and tobacco plants under stress conditions shows that plants emit species-specific ultrasonic distress waves or signals into the air when they experience physical damage. Ultrasound turned out to have this extra sensitivity.

Farmers could hear, or sense, the quiet sounds of dysfunction, in real time, identifying problems before they spread, when they are still reversible. However, while classification tasks such as pest detection often yield high accuracy, the reliability of ultrasound systems in estimating continuous physiological or biochemical variables is more variable. In domains such as animal science and clinical diagnostics, reported R² values for ultrasound-based continuous estimates typically range from 0.33 to 0.52 or lower, reflecting moderate predictive performance under field conditions (Pirmoazen et al., 2020; Bahelka et al., 2009). In agricultural settings, this limitation becomes particularly critical when the goal is to continuously monitor changes in plant hydration, tissue elasticity, or biochemical composition metrics that can vary subtly over time and under different conditions. The performance of such estimations is often affected by system parameters (e.g., frequency, intensity), ambient noise, and the heterogeneity of biological materials, especially in outdoor environments. These findings suggest that although ultrasound is capable of capturing rich internal signatures, its quantitative outputs may fluctuate depending on external and internal factors. Thus, achieving high reliability in continuous-variable estimation requires validated calibration protocols, adaptive signal processing, and possibly multimodal integration with complementary sensing methods.

Ultrasound-based acoustics effectively listen in on insect pests and other hidden threats. Many plant destructive insects, including weevils, borers, and beetle larvae, spend much of their life cycle encased within plant or soil tissues. Still, they generate low audible or ultrasonic sounds (feeding, chewing or movement) that are detectable with ultrasound equipment. Researchers have created listening systems that employ broadband ultrasonic sensors to pick up these sounds and filter them out from ambient noise. For instance, an acoustic ultrasound method was effectively used to record the signature sounds made from the red palm weevil feeding within palm trunks, which enabled the early detection of infestation before there were outward signs on the plant. Other work has employed piezoelectric ultrasound sensors with automated pattern recognition to detect insects chewing within trees, obtaining detection rates around 95% (Hetzroni et al., 2016) for the presence of pests while filtering away other environmental ambient sounds. Wavelet packet transform and machine learning (support vector machines) were used for monitoring cicada pest activity in coffee plantations by a field-deployed acoustic monitoring system, detecting pest activity with over 96% (Escola et al., 2020) accuracy and showing that ultrasound pest surveillance is feasible under high noise outdoor conditions. By detecting infestations early, such ultrasonic pest-detection tools facilitate more targeted interventions (such as localized treatment or quarantine), thereby reducing crop losses.

These novel ultrasound applications hold significant implications in terms of sustainable agriculture. Early detection of problems, whether water stress, disease, or pests, means that farmers can respond precisely, applying water or treatments exactly when and where needed and potentially preventing crop loss or quality degradation. Such precision not only increases yields but also decreases the need for chemical pesticides and fertilizers, bringing agricultural practice closer to environmental sustainability goals by reducing the excess of inputs. Rather than waiting until a field of crops appears physically sick, farmers can get a continuous read on crop health and address potential problems immediately as they arise. Furthermore, ultrasonic devices can be relatively low-cost and portable, making this technology widely available even in resource-limited environments.

The growing integration of artificial intelligence into ultrasound-based monitoring systems is enabling more precise, efficient, and scalable solutions in agriculture. Machine learning algorithms can extract meaningful patterns from complex ultrasound signals and images, facilitating the detection of subtle indicators of plant stress, seed viability, soil conditions, or pest activity. Among the commonly used methods, convolutional neural networks (CNNs) are particularly effective for pattern and image recognition, support vector machines (SVMs) offer reliable classification in smaller datasets, and Long Short-Term Memory (LSTM) networks are well-suited for analyzing temporal changes in acoustic emissions. These AI techniques enhance system responsiveness and reduce the dependence on manual interpretation, allowing real-time insights to be generated even in noisy or dynamic field environments. By combining AI with ultrasound sensing, farmers and researchers can better monitor crop and soil health, optimize resource use, and move closer to data-driven decision-making for sustainable agricultural management.

There are hurdles to be crossed before ultrasound techniques reach their complete potential in precision farming. One primary concern is the interference of ambient noise in real-world field settings. Wind, rain, farm machinery and even animal calls can emit acoustic signals that obscure or simulate the ultrasonic signatures of plant or pest activity. Although sophisticated signal processing (e.g., custom filtering algorithms and machine learning classifiers) has made it possible to suppress some of that noise, detecting biologically relevant signals under all conditions remains challenging, especially when ultrasound is applied in the field. Another obstacle is the lack of standardized protocols and calibration, as varying ultrasonic frequencies, transducer types, and analysis techniques are often used between studies and sensor systems, so direct comparison of results can be difficult. Such fragmentation exposes a critical gap in community-wide standards and shared reference datasets. Adopting consistent methodologies and data formats would enable rapid, robust training of AI models on large, pooled datasets and increase reproducibility of results across labs and crop types. From an engineering perspective, existing ultrasonic devices must be repurposed to meet more challenging field conditions. Sensors must be made more energy efficient, smaller, rugged, and sensitive. There is progression in this regard. More recent designs (micromachined ultrasonic transducer and low-power Internet of Things–connected acoustic sensors) are emerging. However, work remains to ensure that ultrasound systems can run unattended in remote farms with minimal maintenance.

## 5 Open problems and future research directions

In the future, integrating ultrasound with other advanced sensing and analytics capabilities will continue to enhance its potential in precision agriculture. Multi-modal approaches show considerable promise, as ultrasound combined with optical, thermal, or laser-based imaging can provide complementary insights into crop health by capturing internal and external physiological changes. For instance, hybrid acoustic–laser tomography has demonstrated enhanced sensitivity for detecting subsurface structural defects in tree trunks. Integrating ultrasound data with other modalities, supported by AI-driven analysis and IoT frameworks, may ultimately lead to real-time, automated agricultural decision-making systems. The development of standardized measurement protocols and open-access acoustic databases will be critical to enable robust machine learning models capable of generalizing across diverse crops and environmental conditions.

The development direction of the current research indicates that ultrasound will be an increasingly important, sustainable, quantitative agriculture tool and a noninvasive, measuring-based integrated platform. However, several significant challenges and open research issues must be addressed to completely realize ultrasound’s transformative power for agriculture. The challenges are outlined below.

- Environmental Acoustic Noise and Signal Quality: One of the foremost challenges in field applications is the high level of environmental acoustic noise. Influences from wind, rain, and machines bring heavy interference and make weak ultrasound signals reflecting from biological targets difficult to detect. Although the ML-based denoising approaches showed potential, there is a need for real-time, lightweight, and robust noise suppression methods optimized for low SNR conditions, which are typical of outdoor settings.
- Standardization of Ultrasound Measurement Protocols: Standardization of measurement procedures, including calibration, acquisition parameters, and reporting conventions, is essential to ensure the comparability of results across different systems and applications. The current diversity in transducer designs, operating frequencies, and signal processing techniques introduces significant variability, making it challenging to assess key reliability metrics such as accuracy and precision. Furthermore, the lack of uniform benchmarks and the limited reporting of uncertainty margins hinder confidence in quantitative outcomes, particularly for continuous-variable estimations. Developing standardized protocols and open reference datasets would not only enhance reproducibility but also enable more consistent evaluation of model performance, ultimately supporting more trustworthy and actionable decision-making in precision agriculture.
- Sensor Design for Field Deployment: A significant technological gap is the lack of energy-efficient ultrasound sensors that can be left in place for an extended period in agricultural environments. Today, the available systems are developed for laboratory environments but not for field settings. Further work is required to develop field-worthy, low-power ultrasound sensors, potentially drawing upon advances in wireless IoT architectures to form scalable, low-cost monitoring networks.
- Characterization of Plant Materials for Ultrasound Applications: Plant tissues are more heterogeneous and anisotropic compared to soft biological tissues typically analyzed in medical ultrasound. This makes quantitative interpretation of ultrasound signals more challenging, especially under environmental variability. The lack of standardized acoustic property datasets for different crop species adds to the uncertainty in predicting plant water status, elasticity, or stress markers. Additionally, some ultrasound applications require extrapolating signal features into physical or chemical indices (e.g., soluble solids, firmness), which can introduce model uncertainty. Future research should aim at developing calibrated phantoms, reference samples, and *in vivo* datasets that link ultrasound response with precisely measured biological variables to enhance both model training and interpretability.

In addition to these ultrasound-specific challenges, broader enablers such as integration with multimodal sensing systems and the availability of open-access datasets are important for scaling up any smart agricultural technology. While not unique to ultrasound, combining it with other sensing modalities (e.g., hyperspectral or thermal imaging) can improve diagnostic accuracy, especially under noisy or variable field conditions. Similarly, the lack of annotated ultrasound datasets for agriculture limits the development and benchmarking of robust machine learning models. Addressing these systemic needs will benefit the wider agri-tech ecosystem and further support ultrasound adoption.

Beyond technical constraints, the limited adoption of ultrasound systems in agriculture may also stem from economic and behavioral factors. Although several commercial systems (e.g., PiCUS, IoTree) have demonstrated field viability, their penetration into mainstream farming practices remains low compared to optical or infrared-based remote sensing. Factors such as user familiarity, perceived complexity, and cost benefit uncertainty often hinder uptake. Additionally, the lack of service infrastructure, training programs, and agronomic decision support tools integrated with ultrasound data can limit trust and usability among farmers. Unlike visual sensors that provide immediately interpretable images, ultrasound often requires specialized post-processing, which may not appeal to low-resource users. To foster broader adoption, future systems must prioritize user-centered design, affordability, and integration into existing farm management platforms.

Addressing these open challenges will be crucial to fully unlocking ultrasound’s potential as a transformative tool in agriculture. Continued interdisciplinary research combining sensing technologies, machine learning, and plant science will pave the way toward more sustainable, resilient, and data-driven farming systems.

## References

[B1] Abdulsalam S. Alomari Z. Mahmood M. (2023). Ultrafast ultrasound imaging beamforming techniques: A review. Muthanna J. Eng. Technol. 11, 30–35. doi: 10.52113/3/eng/mjet/2023-11-02/30-35

[B2] Agarwal I. Rana D. Shah P. Dude A. Patel P. (2024). “Optimizing crop monitoring: A data warehouse approach in precision agriculture,” in 2024 15th International Conference on Computing Communication and Networking Technologies (ICCCNT), Kamand, India, 1–7. doi: 10.1109/ICCCNT61001.2024.10724191

[B3] Agrawal J. Arafat M. Y. (2024). Transforming farming: A review of AI-powered UAV technologies in precision agriculture. Drones 8, 664. doi: 10.3390/drones8110664

[B4] Agrint Ltd (2024). IoTree Sensor for Red Palm Weevil Detection (Israel: Agrint Ltd.). Available online at: https://www.agrint.net/iotree.

[B5] Ambaru B. Manvitha R. Madas R. (2025). Synergistic integration of remote sensing and soil metagenomics data: Advancing precision agriculture through interdisciplinary approaches. Front. Sustain. Food Syst. 8. doi: 10.3389/fsufs.2024.1499973

[B6] Ashry I. Wang B. Mao Y. Sait M. Guo Y. Al-Fehaid Y.. (2022). CNN–aided optical fiber distributed acoustic sensing for early detection of red palm weevil: A field experiment. Sensors 22, 6491. doi: 10.3390/s22176491, PMID: 36080949 PMC9459888

[B7] Ba M. (2016). Strategic agricultural commodity value chains in africa for increased food: The regional approach for food security. Agric. Sci. 07, 549–585. doi: 10.4236/as.2016.79055

[B8] Bahelka I. Oravcová M. Peskoviˇ covˇ á D. Tomka J. Hanusová E. Lahuckˇ ý R.. (2009). Comparison of accuracy of intramuscular fat prediction in live pigs using five different ultrasound intensity levels. Animal 3, 1205–1211. doi: 10.1017/S1751731109004480, PMID: 22444851

[B9] Barakat M. A. Y. (2023). Fabrication of metal–polymer matching layers to improve some ultrasonic transducers for NDT and calibration. J. Materials Science: Materials Electron. 34, 1031. doi: 10.1007/s10854-023-10458-y

[B10] Baser H. Selvam T. Schwieger W. Hartmann M. (2023). *In situ* ultrasonic measurements as a tool to characterize the stages of the zeolite a crystallization process. Chemie Ingenieur Technik 95, 1824–1833. doi: 10.1002/cite.202300095

[B11] Bente K. Rus J. Mooshofer H. Gaal M. Grosse C. U. (2023). Broadband air-coupled ultrasound emitter and receiver enable simultaneous measurement of thickness and speed of sound in solids. Sensors 23, 1379. doi: 10.3390/s23031379, PMID: 36772419 PMC9919981

[B12] Bonisoli L. Forti L. Arru L. (2025). Outdoor detection of plant ultrasonic emissions using a contactless microphone. Physiologia 5, 9. doi: 10.3390/physiologia5010009

[B13] Bradley S. Ghimire C. (2024). Design of an ultrasound sensing system for estimation of the porosity of agricultural soils. Sensors 24, 2266. doi: 10.3390/s24072266, PMID: 38610477 PMC11014340

[B14] Budiastra I. W. Jannah F. (2022). Non destructive determination of soursop firmness and sweetness with ultrasonic method. Jurnal Teknik Pertanian Lampung (Journal Agric. Engineering) 11, 253–265. doi: 10.23960/jtep-l.v11.i2.253-265

[B15] Chandrarekha C. Guledagudda S. S. Biradar N. Kulkarni G. N. (2024). Impact of agriculture growth on poverty reduction: A case of Karnataka, India. J. Exp. Agric. Int. 46, 91–97. doi: 10.9734/jeai/2024/v46i62460

[B16] Charrier G. Pramsohler M. Charra-Vaskou K. Saudreau M. Améglio T. Neuner G.. (2015). Ultrasonic emissions during ice nucleation and propagation in plant xylem. New Phytol. 207, 570–578. doi: 10.1111/nph.13361, PMID: 25756189 PMC5024006

[B17] Chen C. Pertijs M. A. P. (2021). Integrated transceivers for emerging medical ultrasound imaging devices: A review. IEEE Open J. Solid-State Circuits Soc. 1, 104–114. doi: 10.1109/OJSSCS.2021.3115398

[B18] Cheng D. Y. Liao I. H. Yu J. Liao Y.-C.. (2022). Highly compressible hydrogel reinforced with cellulose nanocrystals for ultrasound scanning via microwave-assisted synthesis. Cellulose 29, 9791–9802. doi: 10.1007/s10570-022-04854-6

[B19] Choi J. Lee D. Son Y. (2021). Ultrasound-assisted soil washing processes for the remediation of heavy metals contaminated soils: The mechanism of the ultrasonic desorption. Ultrasonics Sonochemistry 74, 105574. doi: 10.1016/j.ultsonch.2021.105574, PMID: 33975185 PMC8122358

[B20] da Silva Medeiros D. C. C. Piechontcoski F. da Rocha Watanabe E. R. L. Chaves E. S. Inglez S. D. (2020). Fast and effective simultaneous determination of metals in soil samples by ultrasound-assisted extraction and flame atomic absorption spectrometry: Assessment of trace elements contamination in agricultural and native forest soils from Paraná - Brazil. Environ. Monit. Assess. 192, 111. doi: 10.1007/s10661-020-8065-0, PMID: 31938942

[B21] Dedieu B. Schiavi S. (2019). Insights on work in agriculture. Agron. Sustain. Dev. 39, 56. doi: 10.1007/s13593-019-0601-3

[B22] de Souza U. B. Escola J. P. L. Maccagnan D. H. B. Brito L. d. C. Guido R. C. (2022). Empirical mode decomposition applied to acoustic detection of a cicadid pest. Comput. Electron. Agric. 199, 107181. doi: 10.1016/j.compag.2022.107181

[B23] Ding W. Abdel-Basset M. Alrashdi I. Hawash H. (2024). Next generation of computer vision for plant disease monitoring in precision agriculture: A contemporary survey, taxonomy, experiments, and future direction. Inf. Sci. 665, 120338. doi: 10.1016/j.ins.2024.120338

[B24] Duveiller G. Hammerle A. Hänchen L. Martini D. Migliavacca M. Scholz K.. (2024). Exploring the feasibility of early stress detection with sun-induced chlorophyll fluorescence from tower to satellite. EGU General Assembly, Vienna, Austria (2024) 14–19. doi: 10.5194/egusphere-egu24-12555, 2024.

[B25] Egbokhaebho B. Olalere B. Gidiagba J. Okparaeke J. Awole A. Ehiobu N. (2023). Review on non-destructive techniques for early flaw detection in inspections. Materials Corrosion Eng. Manage. 4, 44–50. doi: 10.26480/macem.02.2023.44.50

[B26] El-Mesery H. S. Mao H. Abomohra A. E.-F. (2019). Applications of non-destructive technologies for agricultural and food products quality inspection. Sensors 19, 846. doi: 10.3390/s19040846, PMID: 30781709 PMC6413199

[B27] Escola J. P. L. Guido R. C. da Silva I. N. Cardoso A. M. Maccagnan D. H. B. Dezotti A. K. (2020). Automated acoustic detection of a cicadid pest in coffee plantations. Comput. Electron. Agric. 169, 105215. doi: 10.1016/j.compag.2020.105215

[B28] Fakopp Enterprise Bt (2023). ArborSonic 3D Acoustic Tomograph (Hungary: Fakopp Enterprise Bt.). Available online at: https://fakopp.com/en/product/arborsonic/.

[B29] Falcioni R. de Oliveira R. B. Chicati M. L. Antunes W. C. Dematte,ˆ J. A. M. Nanni M. R. (2024). Fluorescence and hyperspectral sensors for nondestructive analysis and prediction of biophysical compounds in the green and purple leaves of tradescantia plants. Sensors 24, 6490. doi: 10.3390/s24196490, PMID: 39409529 PMC11479283

[B30] Fariñas M. D. Sancho Knapik D. Peguero Pina J. J. Gil Pelegrin E. Gómez Álvarez-Arenas T. E. (2014). Monitoring plant response to environmental stimuli by ultrasonic sensing of the leaves. Ultrasound Med. Biol. 40, 2183–2194. doi: 10.1016/j.ultrasmedbio.2014.04.004, PMID: 25023117

[B31] Foiret J. Cai X. Bendjador H. Park E.-Y. Kamaya A. Ferrara K. W. (2022). Improving plane wave ultrasound imaging through real-time beamformation across multiple arrays. Sci. Rep. 12, 13386. doi: 10.1038/s41598-022-16961-2, PMID: 35927389 PMC9352764

[B32] Fuentes-Peñailillo F. Gutter K. Vega R. Carrasco Silva G. (2024). Transformative technologies in digital agriculture: Leveraging internet of things, remote sensing, and artificial intelligence for smart crop management. J. Sensor Actuator Networks 13, 39. doi: 10.3390/jsan13040039

[B33] Gómez Álvarez Arenas T. Gil-Pelegrin E. Ealo Cuello J. Fariñas M. D. Sancho-Knapik D. Collazos Burbano D. A.. (2016). Ultrasonic sensing of plant water needs for agriculture. Sensors 16, 1089. doi: 10.3390/s16071089, PMID: 27428968 PMC4970135

[B34] Görres C. M. Chesmore D. (2019). Active sound production of scarab beetle larvae opens up new possibilities for species-specific pest monitoring in soils. Sci. Rep. 9, 10115. doi: 10.1038/s41598-019-46121-y, PMID: 31300666 PMC6626128

[B35] Goswami J. Senpakapriya V. Goswami C. Sarma K. K. Aggarwal S. P. (2024). Disaster preparedness and capacity building for resilience in agriculture. Int. Arch. Photogramm. Remote Sens. Spatial Inf. Sci., XLVIII-5-2024, 17–22. Presented at the ISPRS TC V Mid-term Symposium “Insight to Foresight via Geospatial Technologies. Manila, Philippines 6–8. doi: 10.5194/isprs-archives-XLVIII-5-2024-17-2024

[B36] Grager J.-C. Kotschate D. Gamper J. Gaal M. Pinkert K. Mooshofer H.. (2018). Advances in air-coupled ultrasonic testing combining an optical microphone with novel transmitter concepts. 12th European Conference on Non-Destructive Testing (ECNDT), Gothenburg. ECNDT–E0166. 23(8). Available at: https://www.ndt.net/?id=22756

[B37] Gudra T. (2008). Ultrasounds in gas media: Generation, transmission, applications. Arch. Acoustics 33, 581–592.

[B38] Guo M. Ma Y. Yang X. Mankin R. W. (2019). Detection of damaged wheat kernels using an impact acoustic signal processing technique based on Gaussian modelling and an improved extreme learning machine algorithm. Biosyst. Eng. 184, 37–44. doi: 10.1016/j.biosystemseng.2019.04.022

[B39] Hashemi H. S. Hyun D. Baek J. Natarajan A. Tabesh F. Paulmurugan R.. (2024). “Enhancing ultrasound molecular imaging: RPCA-based filtering to differentiate tumor-bound and free microbubbles,” in 2024 IEEE Ultrasonics, Ferroelectrics, and Frequency Control Joint Symposium (UFFC-JS) (Taipei, Taiwan), 1–6. doi: 10.1109/UFFC-JS60046.2024.10793633

[B40] He Y. Wan H. Jiang X. Peng C. (2023). Piezoelectric micromachined ultrasound transducer technology: Recent advances and applications. Biosensors 13, 55. doi: 10.3390/bios13010055, PMID: 36671890 PMC9856188

[B41] Hetzroni A. Soroker V. Cohen Y. (2016). Toward practical acoustic red palm weevil detection. Comput. Electron. Agric. 124, 100–106. doi: 10.1016/j.compag.2016.03.018

[B42] Hossain M. M. Sultana F. Mostafa M. Ferdus H. Rahman M. Rana J. A.. (2024). Plant disease dynamics in a changing climate: Impacts, molecular mechanisms, and climate-informed strategies for sustainable management. Discover Agric. 2, 132. doi: 10.1007/s44279-024-00144-w

[B43] Huang K. Li Q. Zhu K. Chen B. Qian X. Wang X.. (2023). A through-transmission ultrasonic method for the detection of ferrite tile defects. Appl. Sci. 13, 11172. doi: 10.3390/app132011172

[B44] IML Electronic GmbH (2021). PiCUS Sonic Tomograph (Germany: IML Electronic GmbH). Available online at: https://www.iml-electronic.com/product/picus-sonic-tomograph/.

[B45] Jiang C. Li Z. Zhang Z. Wang S. (2023). A new design to Rayleigh wave EMAT based on spatial pulse compression. Sensors 23, 3943. doi: 10.3390/s23083943, PMID: 37112283 PMC10146980

[B46] Jin Y. Y. Gao W. L. Zhang H. An D. Guo S. H. Ahmed S. I.. (2016). Identification of damaged corn seeds using air-coupled ultrasound. Int. J. Agric. Biol. Eng. 9, 63–70. doi: 10.3965/j.ijabe.20160901.1880

[B47] Joshi S. R. Rakesh U. Sripad S. (2024). “Dual sensor dynamics: A comparative analysis of plant health assessment,” in 2024 3rd International Conference on Power Electronics and IoT Applications in Renewable Energy and its Control (PARC), Mathura, India, 255–260. doi: 10.1109/PARC59193.2024.10486390

[B48] Kewalramani J. A. Wang B. Marsh R. W. Meegoda J. N. Rodriguez Freire L. (2022). Coupled high and low-frequency ultrasound remediation of PFAS-contaminated soils. Ultrason. Sonochem. 88, 106063. doi: 10.1016/j.ultsonch.2022.106063, PMID: 35738199 PMC9218828

[B49] Khait I. Lewin-Epstein O. Sharon R. Saban K. Goldstein R. Anikster Y.. (2023). Sounds emitted by plants under stress are airborne and informative. Cell 186, 1328–1336.e10. doi: 10.1016/j.cell.2023.03.009, PMID: 37001499

[B50] Kidav J. Pillai P. M. Deepak V. Sreejeesh S. G.. (2022). Design of a 128-channel transceiver hardware for medical ultrasound imaging systems. IET Circuits Devices Syst. 16, 92–104. doi: 10.1049/cds2.12087

[B51] Kilinc E. Orhan U. (2025). Improving the accuracy of soil texture determination using pH and electro conductivity values with ultrasound penetration-based digital soil texture analyzer. PeerJ Comput. Sci. 11, e2663. doi: 10.7717/peerj-cs.2663, PMID: 40062284 PMC11888846

[B52] Koulountzios P. Rymarczyk T. Soleimani M. (2019). A quantitative ultrasonic travel-time tomography to investigate liquid elaborations in industrial processes. Sensors 19, 5117. doi: 10.3390/s19235117, PMID: 31766718 PMC6928607

[B53] Krautkrämer J. Krautkrämer H. (1990). Ultrasonic Testing of Materials. 4 edn (Berlin Heidelberg: Springer-Verlag). doi: 10.1007/978-3-662-10680-8

[B54] Li X. Yin J. Hu C. Zhou Q. Shung K. K. Chen Z. (2010). High-resolution coregistered intravascular imaging with integrated ultrasound and optical coherence tomography probe. Appl. Phys. Lett. 97, 133702. doi: 10.1063/1.3493659, PMID: 20981274 PMC2962660

[B55] Liang H. Song C. Wang R. Yang H. (2023). A model for multiphase flow velocity calculation in pipelines based on ultrasonic sensors. Phys. Fluids 35, 093308. doi: 10.1063/5.0165640

[B56] Liu L. Abdulla W. (2023). Improving APT systems’ performance in air via impedance matching and 3D-printed clamp. Sensors 23, 5347. doi: 10.3390/s23115347, PMID: 37300074 PMC10255979

[B57] Lluveras Núñez D. Molero-Armenta M. García Izquierdo M. González Hernández M. Anaya Velayos J. J. (2017). Ultrasound transmission tomography for detecting and measuring cylindrical objects embedded in concrete. Sensors 17, 1085. doi: 10.3390/s17051085, PMID: 28489062 PMC5470475

[B58] Logeshwaran J. Srivastava D. Kumar K. S. Rex M. J. Al-Rasheed A. Getahun M.. (2024). Improving crop production using an agro-deep learning framework in precision agriculture. BMC Bioinf. 25, 341. doi: 10.1186/s12859-024-05970-9, PMID: 39487390 PMC11529011

[B59] Lu H. Cui H. Lu G. Jiang L. Hensleigh R. Zeng Y.. (2023). 3D printing and processing of miniaturized transducers with near-pristine piezoelectric ceramics for localized cavitation. Nat. Commun. 14, 2418. doi: 10.1038/s41467-023-37335-w, PMID: 37105973 PMC10140030

[B60] Luca R. Forzoni L. Gelli F. Bamber J. (2021). An educational overview of ultrasound probe types and their fields of application. Arch. Acoustics 46, 3–15. doi: 10.24425/aoa.2021.136555

[B61] Lyu F. Zhou X. Ding Z. Qiao X. Song D. (2024). Application research of ultrasonic-guided wave technology in pipeline corrosion defect detection: A review. Coatings 14, 358. doi: 10.3390/coatings14030358

[B62] Maginga T. J. Masabo E. Bakunzibake P. Kim K. S. Nsenga J. (2024). Using wavelet transform and hybrid CNN–LSTM models on VOC & ultrasound IoT sensor data for non-visual maize disease detection. Heliyon 10, e26647. doi: 10.1016/j.heliyon.2024.e26647, PMID: 38420424 PMC10901083

[B63] Mahanti N. K. Pandiselvam R. Kothakota A. Ishwarya P. S. Chakraborty S. K. Kumar M.. (2022). Emerging non-destructive imaging techniques for fruit damage detection: Image processing and analysis. Trends Food Sci. Technol. 120, 418–438. doi: 10.1016/j.tifs.2021.12.021

[B64] Mankin R. W. Al-Ayedh H. Y. Aldryhim Y. Rohde B. (2016). Acoustic detection of Rhynchophorus ferrugineus (Coleoptera: Dryophthoridae) and Oryctes elegans (Coleoptera: Scarabaeidae) in Phoenix dactylifera (Arecales: Arecacae) trees and offshoots in Saudi Arabian orchards. J. Economic Entomology 109, 622–628. doi: 10.1093/jee/tov398, PMID: 26743218

[B65] Mankin R. W. Hagstrum D. W. Smith M. T. Roda A. L. Kairo M. T. K. (2011). Perspective and promise: A century of insect acoustic detection and monitoring. Am. Entomologist 57, 30–44. doi: 10.1093/ae/57.1.30

[B66] Martin B. Shaby S. M. Premi M. G. (2015). Studies on acoustic activity of red palm weevil the deadly pest on coconut crops. Proc. Materials Sci. 10, 455–466. doi: 10.1016/j.mspro.2015.06.081

[B67] Martynenko A. V. Ermachenko V. P. (2021). On the question of interpreting the echograms of ultrasonic pulse flaw detector. Russian J. Nondestructive Testing 57, 656–668. doi: 10.1134/S1061830921080064

[B68] Miraei Ashtiani S. H. Salarikia A. Golzarian M. R. Emadi B. (2016). Non-destructive estimation of mechanical and chemical properties of persimmons by ultrasonic spectroscopy. Int. J. Food Properties 19, 1522–1534. doi: 10.1080/10942912.2015.1082485

[B69] Nanda M. A. Seminar K. B. Maddu A. Nandika D. (2023). Acoustic and temperature signals generated by subterranean termite infestation: Its characteristics and implementations. All Life 16. doi: 10.1080/26895293.2023.2167866

[B70] Narumanchi V. V. Pourahmadian F. Lum J. Townsend A. Tringe J. W. Stobbe D. M.. (2023). Laser ultrasonic imaging of subsurface defects with the linear sampling method. Optics Express 31, 9098–9111. doi: 10.1364/OE.485084, PMID: 36860009

[B71] Nasir G. Zaidi S. Ahmad S. Asfaq F. M. Allai Ahmad F.. (2024). Current status of technological advancement of ultrasound processing in the food industry and its SWOT analysis. Crit. Rev. Food Sci. Nutr., 1–18. doi: 10.1080/10408398.2024.2405992, PMID: 39350537

[B72] Oates C. (2023). The Basic Physics of Ultrasound Vol. chap. 1 (Chichester, West Sussex, UK: John Wiley & Sons, Ltd), 5–23. doi: 10.1002/9781119891581.ch1

[B73] Orhan U. Kilinc E. Albayrak F. Aydin A. A. Torun A. (2022). Ultrasound penetration-based digital soil texture analyzer. Arabian J. Sci. Eng. 47, 10751–10767. doi: 10.1007/s13369-022-06766-w

[B74] Pandi M. D. Shridar B. (2017). Assessment of economic losses due to agricultural accidents in selected districts of Tamil Nadu, India. Int. J. Curr. Microbiol. Appl. Sci. 6, 1132–1139. doi: 10.20546/ijcmas.2017.612.127

[B75] Patel R. Patel D. Meshram D. (2022). A review on non-destructive testing (NDT) techniques: Advances, researches and applicability. Internat. J. Curr. Sci. Res. Rev. 5, 1342–1355. doi: 10.47191/ijcsrr/V5-i4-59

[B76] Pinhas J. Soroker V. Hetzroni A. Mizrach A. Teicher M. Goldberger J. (2008). Automatic acoustic detection of the red palm weevil. Comput. Electron. Agric. 63, 131–139. doi: 10.1016/j.compag.2008.02.004

[B77] Piriyadharshini S. Ezhilarasi P. (2023). “A case study on non-invasive plant health diagnosis using multi-view stereo spectral imaging,” in Proceedings of the 2023 International Conference on Recent Methods in Knowledge Management and Technology Enhancement (RMKMATE), Chennai, India, 1–7. doi: 10.1109/RMKMATE59243.2023.10369333

[B78] Pirmoazen A. M. Khurana A. El Kaffas A. Kamaya A. (2020). Quantitative ultrasound approaches for diagnosis and monitoring hepatic steatosis in nonalcoholic fatty liver disease. Theranostics 10, 4277–4289. doi: 10.7150/thno.40249, PMID: 32226553 PMC7086372

[B79] Plense Technologies (2024). Ultrasound Sensors for Plant Health Monitoring (Netherlands: Plense Technologies). Available online at: https://plense.tech.

[B80] Pusppanathan J. Satria M. H. Rahim R. A. Mohd E. J. Nor Ayob N. M. Leow P. L.. (2017). Finite element analysis for acoustic wave transmission in ultrasonic tomography application. Int. J. Integrated Eng. 9, 43–48. Available at: https://publisher.uthm.edu.my/ojs/index.php/ijie/article/view/2014

[B81] Qiu Q. Qin R. Lam J. H. Tang A. M. Leung M. W. Lau D. (2019). An innovative tomographic technique integrated with acoustic-laser approach for detecting defects in tree trunk. Comput. Electron. Agric. 156, 129–137. doi: 10.1016/j.compag.2018.11.017

[B82] Rashmi M. R. Gopalakrishnan M. Rajeswari R. Senthil K. M. Kavitha M. Maragatham S. (2024). Resilient soils for a changing climate: Navigating the future of sustainable agriculture. Plant Sci. Today 11, 01–12. doi: 10.14719/pst.5598

[B83] Ricci M. Barbi E. Dimitri M. Duranti C. Arcangeli A. Corvi A. (2024). Sonoporation, a novel frontier for cancer treatment: A review of the literature. Appl. Sci. 14, 515. doi: 10.3390/app14020515

[B84] Rinntech GmbH (2022). Arbotom Sonic Tomograph (Germany: Rinntech GmbH).

[B85] Sennoga C. A. (2020). “Ultrasound imaging,” in Bioengineering Innovative Solutions for Cancer, vol. chap. 2.2 . Eds. Ladame S. Chang J. Y. (Cambridge, MA, USA: Academic Press), 123–161. doi: 10.1016/B978-0-12-813886-1.00007-3

[B86] Singh A. K. Pathak M. Joshi M. D. Kumar S. Kashyap S. Hasan W. (2024). The role of agriculture in poverty alleviation and rural development. J. Sci. Res. Rep. 30, 529–549. doi: 10.9734/jsrr/2024/v30i82276

[B87] Singh J. Tant K. Curtis A. Mulholland A. (2022). Real-time super-resolution mapping of locally anisotropic grain orientations for ultrasonic non-destructive evaluation of crystalline material. Neural Computing Appl. 34, 4993–5010. doi: 10.1007/s00521-021-06670-8

[B88] Sinha P. Kathare P. Roy A. (2023). Recent advances for detecting and addressing plant disease: Towards future farming. Int. J. Environ. Climate Change 13, 87–94. doi: 10.9734/ijecc/2023/v13i113148

[B89] Soltani Firouz M. Farahmandi A. Hosseinpour S. (2021). Early detection of freeze damage in navel orange fruit using nondestructive low intensity ultrasound coupled with machine learning. Food Anal. Methods 14, 1140–1149. doi: 10.1007/s12161-020-01942-w

[B90] Song J. Xue C. He C. Zhang R. Mu L. Cui J.. (2015). Capacitive micromachined ultrasonic transducers (cmuts) for underwater imaging applications. Sensors 15, 23205–23217. doi: 10.3390/s150923205, PMID: 26389902 PMC4610594

[B91] Su Q. Tan C. Dong F. (2017). Measurement of oil–water two-phase flow phase fraction with ultrasound attenuation. IEEE Sensors J. 18, 1150–1159. doi: 10.1109/JSEN.2017.2779868

[B92] Toffessi Siewe S. Callé S. Vander Meulen F. Valente D. Grégoire J.-M. Banquart A.. (2023). High acoustic impedance and attenuation backing for high-frequency focused p(vdf-trfe)-based transducers. Sensors 23, 4686. doi: 10.3390/s23104686, PMID: 37430599 PMC10223069

[B93] Vasighi-Shojae H. Gholami-Parashkouhi M. Mohamadzamani D. Soheili A. (2020). Predicting mechanical properties of golden delicious apple using ultrasound technique and artificial neural network. Food Anal. Methods 13, 699–705. doi: 10.1007/s12161-019-01689-z

[B94] Vasighi-Shojae H. Gholami-Parashkouhi M. Mohammadzamani D. Soheili A. (2018). Ultrasonic based determination of apple quality as a nondestructive technology. Sens. Bio-Sensing Res. 21, 22–26. doi: 10.1016/j.sbsr.2018.09.002

[B95] Vergeynst L. L. Sause M. G. R. Hamstad M. A. Steppe K. (2015). Deciphering acoustic emission signals in drought stressed branches: The missing link between source and sensor. Front. Plant Sci. 6. doi: 10.3389/fpls.2015.00494, PMID: 26191070 PMC4488601

[B96] Vrdoljak I. Dravinac L. (2025). The role of ultrasound in medical diagnostics and treatment. ENTRENOVA - ENTerprise Res. Innovation 10, 163–175. doi: 10.54820/entrenova-2024-0015

[B97] Wang Z. Lu C. Li H. Wang C. Wang L. Yang H. (2024b). Propagation laws of ultrasonic continuous signals at the transmitting transducer–soil interface. Agriculture 14, 1470. doi: 10.3390/agriculture14091470

[B98] Wang B. Mao Y. Ashry I. Al-Fehaid Y. Al-Shawaf A. Ng T. K.. (2021). Towards detecting red palm weevil using machine learning and fiber optic distributed acoustic sensing. Sensors 21, 1592. doi: 10.3390/s21051592, PMID: 33668776 PMC7956387

[B99] Wang J.-x. Sui M.-l. Yang S.-f. Gao X.-w. (2011). “The xylem cavitation diagnosis technology based on ultrasonic acoustic emission,” in 2011 Symposium on Piezoelectricity, Acoustic Waves and Device Applications (SPAWDA), Shenzhen, China,, 581–584. doi: 10.1109/SPAWDA.2011.6167318

[B100] Wang J. Wang Y. Li G. Qi Z. (2024a). Integration of remote sensing and machine learning for precision agriculture: A comprehensive perspective on applications. Agronomy 14, 1975. doi: 10.3390/agronomy14091975

[B101] Wei J. Sun L. Peng C. Fan L. Teng F. Hao W.. (2024). Design and implementation of a digital smart layer for ultrasonic-guided wave-based structural health monitoring. Struct. Health Monit. 0. doi: 10.1177/14759217241295363

[B102] Wells P. Liang H. (2011). Medical ultrasound: Imaging of soft tissue strain and elasticity. J. R. Soc. Interface 8, 1521–1549. doi: 10.1098/rsif.2011.0054, PMID: 21680780 PMC3177611

[B103] Wiskin J. Malik B. Natesan R. Lenox M. (2019). Quantitative assessment of breast density using transmission ultrasound tomography. Med. Phys. 46, 2610–2620. doi: 10.1002/mp.13503, PMID: 30893476 PMC6618090

[B104] Woo D. K. Do W. Hong J. Choi H. (2022). A novel and non-invasive approach to evaluating soil moisture without soil disturbances: Contactless ultrasonic system. Sensors 22, 7450. doi: 10.3390/s22197450, PMID: 36236548 PMC9571307

[B105] Wu D. (2024). “Detecting plant stress and phenotyping in live california crops using thermal imaging technique,” in Proceedings of the 2024 International Conference on Sustainable Smart Agriculture (ICSSA), Penang, Malaysia, 1–6. doi: 10.1109/ICSSA62312.2024.10788629

[B106] Xing Y. Wang X. (2024). Precise application of water and fertilizer to crops: Challenges and opportunities. Front. Plant Sci. 15. doi: 10.3389/fpls.2024.1444560, PMID: 39711591 PMC11659019

[B107] Xu K. Huang M. Xu C. Zhen J. Jin G. Gong H. (2023). Assessment of the bio-cementation effect on shale soil using ultrasound measurement. Soils Foundations 63, 101249. doi: 10.1016/j.sandf.2022.101249

[B108] Xu S. Wang H. Liang X. Lu H. (2024). Research progress on methods for improving the stability of non-destructive testing of agricultural product quality. Foods 13, 3917. doi: 10.3390/foods13233917, PMID: 39682989 PMC11640820

[B109] Yan J. Zhang Y. Jiao Z. Song L. Wang Z. Zhang Q.. (2024). Opportunities and challenges of ultrasonic diagnostic techniques for plant-based food monitoring: Principle, machine system, and application strategies. Crit. Rev. Food Sci. Nutr., 1–20. doi: 10.1080/10408398.2024.2418891, PMID: 39450774

[B110] Yanez J. Uranga A. Barniol N. (2022). Fluid compressional properties sensing at microscale using a longitudinal bulk acoustic wave transducer operated in a pulse-echo scheme. Sensors Actuators A: Phys. 334, 113334. doi: 10.1016/j.sna.2021.113334

[B111] Yang T. Jin Y. Dahotre N. B. Neogi A. (2022). Non-contacting plant health monitoring via ultrasound in ambient air. Biophysica 2, 315–323. doi: 10.3390/biophysica2040029

[B112] Yared H. D. (2011). The possibility of utilizing the normal incidence reflection coefficient of acoustic waves to characterize and study porous granular layers (Stockholm, Sweden: Master’s thesis, KTH, Highway and Railway Engineering).

[B113] Yu K. Zhao S. Sun B. Jiang H. Hu L. Xu C.. (2025). Enhancing food production through modern agricultural technology. Plant Cell Environ. 2025, 1–12. doi: 10.1111/pce.15299, PMID: 39627956

[B114] Yuan J. Li Z. Ma Q. Li J. Li Z. Zhao Y.. (2023). Noninvasive fluid bubble detection based on capacitive micromachined ultrasonic transducers. Microsystems Nanoengineering 9, 20. doi: 10.1038/s41378-023-00491-6, PMID: 36844939 PMC9946994

[B115] Zhang Y. Cegla F. (2022). Co-located dual-wave ultrasonics for component thickness and temperature distribution monitoring. Struct. Health Monit. 22, 1090–1104. doi: 10.1177/14759217221104463

[B116] Zhang X. Cheng H. Xu K. Ding D. Wang X. Wang B.. (2024). Ultrasonic enhancement for mineral flotation: Technology, device, and engineering applications. Minerals 14, 986. doi: 10.3390/min14100986

[B117] Zhang C. Huang W. Liang X. He X. Tian X. Chen L.. (2022). Slight crack identification of cottonseed using air-coupled ultrasound with sound to image encoding. Front. Plant Sci. 13. doi: 10.3389/fpls.2022.956636, PMID: 36186064 PMC9520625

[B118] Zhang T. Unger K. Maire G. Chaumet P. C. Talneau A. Godhavarti C.. (2018). Multiwavelength multi-angle reflection tomography. Optics Express 26, 26093–26105. doi: 10.1364/OE.26.026093, PMID: 30469701

[B119] Zhang H. Wang L. Jin X. Bian L. Ge Y. (2023). High-throughput phenotyping of plant leaf morphological, physiological, and biochemical traits on multiple scales using optical sensing. Crop J. 11, 1303–1318. doi: 10.1016/j.cj.2023.04.014

[B120] Zheng Y. Zhang Z. Zhang Y. Pan Q. Yan X. Li X.. (2024). Enhancing ultrasound power transfer: Efficiency, acoustics, and future directions. Advanced Materials, 2407395. doi: 10.1002/adma.202407395, PMID: 39044603 PMC12160699

[B121] Zhong H. Zhang L. Shi Y. Xia Z. Duan J. Shi B.. (2022). Research on flaw detection experiment of ultrason. J. Physics: Conf. Ser. 2285, 12038. doi: 10.1088/1742-6596/2285/1/012038

